# Oncosuppressive Suicide Gene Virotherapy “PVH1-yCD/5-FC” for Pancreatic Peritoneal Carcinomatosis Treatment: NFκB and Akt/PI3K Involvement

**DOI:** 10.1371/journal.pone.0070594

**Published:** 2013-08-14

**Authors:** Soukaina Réjiba, Christelle Bigand, Celine Parmentier, Ahmed Masmoudi, Amor Hajri

**Affiliations:** 1 Biologie des tumeurs, Université de Strasbourg, Strasbourg, France; 2 Institut Supérieur de Biotechnologie-Sidi Thabet, Tunis, Tunisie; 3 Molecular Biology and Protein Engineering for Cancer Therapy, BioIII Department, FRIAS -University of Freiburg, Freiburg, Germany; Mie University Graduate School of Medicine, Japan

## Abstract

Peritoneal carcinomatosis is common in advanced pancreatic cancer. Despite current standard treatment, patients with this disease until recently were considered incurable. Cancer gene therapy using oncolytic viruses have generated much interest over the past few years. Here, we investigated a new gene directed enzyme prodrug therapy (GDEPT) approach for an oncosuppressive virotherapy strategy using parvovirus H1 (PV-H1) which preferentially replicates and kills malignant cells. Although, PV-H1 is not potent enough to destroy tumors, it represents an attractive vector for cancer gene therapy. We therefore sought to determine whether the suicide gene/prodrug system, yCD/5-FC could be rationally combined to PV-H1 augmenting its intrinsic oncolytic activity for pancreatic cancer prevention and treatment. We showed that the engineered recombinant parvovirus rPVH1-yCD with 5-FC treatment increased significantly the intrinsic cytotoxic effect and resulted in potent induction of apoptosis and tumor growth inhibition in chemosensitive and chemoresistant cells. Additionally, the suicide gene-expressing PV-H1 infection reduced significantly the constitutive activities of NFκB and Akt/PI3K. Combination of their pharmacological inhibitors (MG132 and LY294002) with rPVH1-yCD/5-FC resulted in substantial increase of antitumor activity. *In vivo*, high and sustained expression of NS1 and yCD was observed in the disseminated tumor nodules and absent in normal tissues. Treatment of mice bearing intraperitoneal pancreatic carcinomatosis with rPVH1-yCD/5-FC resulted in a drastic inhibition of tumor cell spreading and subsequent increase in long-term survival. Together, the presented data show the improved oncolytic activity of wPV-H1 by yCD/5-FC and thus provides valuable effective and promising virotherapy strategy for prevention of tumor recurrence and treatment. In the light of this study, the suicide gene parvovirotherapy approach represents a new weapon in the war against pancreatic cancer. Moreover, these preliminary accomplishments are opening new field for future development of new combined targeted therapies to have a meaningful impact on advanced cancer.

## Introduction

Pancreatic cancer is one of the leading causes of cancer-related deaths worldwide [1]. Prognosis of this disease remains disappointing due to its late diagnosis, low surgical resectability rates, and notorious resistance to standard chemotherapy and radiation [2], [3]. Peritoneal carcinomatosis is a frequent cause of death in patients with advanced pancreatic carcinoma. Cytoreductive surgery and conventional treatment strategy has been generally believed to have only a small impact on patients with peritoneal dissemination. Further therapeutic approaches are needed in addition to conventional chemotherapy for this devastating disease. Key challenges facing cancer therapy are the development of tumor-specific drugs and the implementation of potent multimodal treatment regimens.

During the last years, oncolytic virotherapy has entered clinical experimentation [4]–[6]. Some oncolytic viruses are naturally occurring, whilst others are genetically engineered to reduce pathogenicity, enhance tumor cell selectivity and encode therapeutic genes. Many viruses have been engineered or selected to create replicating oncolytic therapeutics that precisely target genetic defects arising during tumor development or unique features. Restriction of viral replication is one of the most used approaches to acquire tumor selectivity. This can be achieved by deletion of specific viral genes, such as the E1B-55kD gene in the ONYX-015 (*dl*1520) adenovirus [5]–[7] or by use of tissue- or tumor-specific promoters [8]–[10].

Although both many naturally occurring and engineered oncotropic viruses have been demonstrated to display specific oncolytic activity, there are few examples of complete tumor eradication. Among different oncolytic viruses, autonomously replicating parvoviruses such as parvovirus H1 (PV-H1) can infect numerous animal species including humans [11], [12] without induction of significant inflammatory reactions [13] or clinical symptoms [14], [15]. The parvoviruses are small, non-enveloped, icosahedral viruses containing linear, single-stranded DNA genomes of about 5 kb composed of two or three open reading frames.

PV-H1 is organized in two overlapping transcription units controlled by the two P4 and P38 promoters. The early P4 promoter directs synthesis of the NS1/NS2 non-structural proteins, while the late P38 promoter regulates the expression of VP1/VP2 capsid proteins. NS1 is a multifunctional nuclear phosphoprotein involved in the transactivation of P38 promoter [16], intervenes in viral replication, and is also responsible for the parvovirus cytotoxicity and lytic activity [17], [18]. Thus, NS1 plays critical roles during parvovirus replication, interaction with target gene expression and induction of cytotoxic activity. It was reported that parvoviruses can inhibit at least partially, the development of spontaneous as well as chemically or virally induced tumors in laboratory animals [19]. The relative oncotropic and oncolytic properties related to the preferential infection and replication of PV-H1 in cancerous or transformed cells make it as a potential candidate for vectors in cancer gene therapy. However, evidently the “wild-type” or the naturally parvovirus infections are not enough potent to prevent cancer or to abolish established tumors. Thus, our approach was to exploit the inherent tumor tropism of parvovirus and to enhance its intrinsic antitumor property. To this end, we choose genetic engineering of recombinant PV-H1 expressing GDEPT system creating a recombinant parvovirus-based vector, in which the capsid proteins VP1/2 were replaced by the cDNA sequence encoding yeast cytosine deaminase (yCD), a suicide gene converting the nontoxic prodrug 5-fluorocytosine (5-FC) into the widely used chemotherapeutic agent 5-fluorouracil (5-FU). The recombinant parvoviral vector hold all the elements involved in the oncotropism (DNA replication origins, P4 promoter, NS proteins) and the oncolytic activity (NS proteins) of the parental viruses. The use of yCD/5-FC suicide gene/prodrug system is particularly remarkable regarding the higher generation of 5-FU within tumor cells and induction of strong bystander effect related to its free diffusion into neighboring untransduced cells not expressing the foreign enzyme [20], [21].

It is well demonstrated that the majority of pancreatic cancers rapidly developed a subclass of refractory tumors due to the induction of resistance mechanisms after chemotherapy treatment. These tumors are particularly characterized by high overexpression or constitutive activity of different transduction pathway signaling mediators. It was reported that PV-H1 induces its cytotoxic and oncolytic activity through a cross talk with some intracellular signals and transcription factors. In the present work, we focused our analysis on PI3K/Akt and NFκB pathways which were extensively studied and seem to be deregulated in many transformation and chemoresistance processes [22], [23]. In certain cases, NFκB is activated during viral infection and interpreted as a protective response of the host [24], [25]. Moreover, the NFκB prosurvival and proliferative activities are often associated to chemoresistance [26], [27] and its regulation, processing, and disruption are being explored as chemotherapeutic targets in cancer [28], [29]. Indirect activation of NFκB pathway due to aberrant oncogenic signaling is a common phenomenon in many types of cancer, which increases the capacity for tumor cells to evade apoptosis and gain a survival advantage over normal, untransformed cells. Concerning the PI3K/Akt pathway signaling, its role in virus infection is not completely clear. However, many examples showed that these mediators are involved in tumor growth and chemoresistance induction [22], [30]. Then, we first asked if the PV-H1 and recombinant derivatives can be confronted to similar molecular resistance mechanisms. Second, we wanted to analyze the effect of PV infections on these specific pathway signals. Thus, we investigated NFκB and PI3K/Akt expression and we asked whether they influence the susceptibility of tumor cells to PV-H1 induced cytotoxicity. To achieve our objectives and demonstrate that this GDEPT parvovirotherapy strategy “PVH1-yCD/5-FC” is efficient in different subtypes of pancreatic adenocarcinoma, this study was performed using BxPc3 chemosensitive and Panc1/Aspc1 chemoresistant pancreatic tumor cell models [31]–[33].

We demonstrated the higher effectiveness of the recombinant rPV-H1/yCD plus 5-FC when compared to its wild-type replicative counterpart (wtPV-H1) *in vitro* and *in vivo*. Additionally, the PV-H1 infection was in correlation with a reduction of NFκB and PI3K/Akt activity. These observations suggest that NFκB and PI3K/Akt inhibitors could increase the anti-tumor activity of PV-H1 and derivatives.

## Materials and Methods

### Cell culture and reagents

Human BxPc3 and AsPc1 pancreatic cell lines were maintained in RPMI 1640-Glutamax. The human pancreatic tumor cell lines, Panc1, the SV40-T-antigen-transformed Hek293T and the NBK newborn kidney cell lines (purchased from ATCC) were grown in DMEM-Glutamax medium-high glucose. Media were supplemented with 10% heat-inactivated fetal calf serum, penicillin (100 U/ml) and streptomycin (100 µg/ml). Cells were maintained at 37°C with 5% CO2. All cell culture products were purchased from Invitrogen-Life Technologies (Cergy Pontoise, France). MG132 and LY294002 were purchased from Sigma-Aldrich (Courtaboeuf, France).

### Recombinant PV-H1 virus constructions for yCD and GFP expression

The PV-H1-based vector DNA, phH1Δ800 [34], was linearized with SmaI restriction enzyme and subsequently dephosphorylated by the calf intestinal phosphatase (New England Biolabs-OZYME, Saint Quentin Yveline; France). The yCD gene was isolated from yeast genomic DNA (strain D4916 from Sigma-Aldrich) using specific probes: Forward: 5′-attctcgagc**gccaccatgg**tgacagggggaatg-3′ containing a Kozak sequence (bold type) and XhoI restriction site (underlined), and Reverse: 5′-attggatccctactcaccaatatcttcaaacc-3′ containing BamH1 restriction site (underlined), and was phosphorylated with T4 Polynucleotide Kinase (New England Biolabs-Ozyme, Montigny-Le-Bretonneux; France). The GFP coding sequence was recovered from pEGFP-N1 plasmid (Clontech, Ozyme,) and submitted to a klenow reaction (Fermentas-Euromedex, Strasbourg; France). Inserts were ligated into the Sma1 site of phH1Δ800 and the two rH1-yCD and rH1-GFP recombinant viruses were grown in *rec*BC *sbc*B *rec*F *Escherichia Coli* strain SURE (Stratagene; France). The resulting recombinant parvoviral plasmids were verified by restriction enzymes and PCR. Then, the selected clones were sequenced and confirmed by the comparison to the published sequences.

### Parvovirus amplification and titration

To produce recombinant parvoviruses, Hek293T cells were cotransfected with 6 µg of rPVH1-yCD/rPVH1-GFP plasmids and 12 µg of PBK helper plasmid using a standard calcium phosphate precipitation method. The helper construct pBK-CMV/VP contains the H1 virus genes encoding the capsid proteins VP1 and VP2 under the control of the immediate-early promoter of human cytomegalovirus [35]. Three days post-transfection, cells were scraped, washed in PBS and resuspended in 50 mM Tris, 0.5 mM EDTA pH 8.7. Virus was released by five rounds of freeze/thawing and purified by ultracentrifugation using Iodixanol gradient.

Recombinant viruses were titrated by infected cells hybridization assays on NBK indicator cells, as described by Maxwell and Maxwell [36]. Infected NBK cells were transferred on nitrocellulose membrane filters. DNA was denaturated with 0.5M NaOH, 1.5M NaCl, neutralized with 1.5M NaCl, 0.5M Tris-HCl (pH 7.2), 1M EDTA, and immobilized 2 h at 80°C in a dried atmosphere. Next, DNA was pre-hybridized for 1 hour at 65°C in presence of sheared-salmon sperm DNA (200 µg/ml), and hybridized for 18 hours at 65°C in a solution containing ^32^P-labeled NS1-specific DNA probes (Mega-Prime DNA labeling Kit, Amersham Biosciences, France). After washings, radioactivity detection and quantification were performed using the PhosphorImager system (Molecular Dynamics, France). Recombinant virus titers were determined and expressed as replication units per milliliter of virus suspension (RU/ml).

### Real-time quantitative RT-PCR

Total RNA was extracted from frozen tumor and matched normal tissues using TRIzol reagent (Invitrogen, Paris, France) in accordance with the manufacturer's instructions. First-strand cDNA was synthesized from total RNA using random hexamer primers and the SuperScript II system for RT-PCR (Invitrogen). Expression analysis for *NS1, GFP and yCD* mRNAs was measured by real-time QRT-PCR using the iQSYBR Green Supermix reagent and MJ Chromo4 Real-Time PCR Detection System (Bio-Rad, Les Ulis, France). Data analysis was performed using Opticon Monitor Analysis Software V3.01 (MJ Research). The expression of each gene was normalized to GAPDH as a reference, and relative levels were calculated from a 4-point standard curve. Independent experiments were performed in triplicate.

The specific primers were: Forwards: 5′-ccacactcaaagagttggtacataa-3′, Reverse: 5′-cacctggttgagccatcat -3′ for NS1, Forwards: 5′-aatggcaagcaagtggggat-3′, Reverse: 5′-cttcaaaccaatcctgaggtc-3′ for yCD, Forwards: 5′-atggacgatctgtttcccct-3′, Reverse: 5′-cggtttactcggcagatctt-3′ for NFκB and Forwards: 5′-accacagtccatgccatcac-3′, Reverse: 5′-tccaccaccctgttgctgta-3′ for GAPDH. The conditions for GAPDH, yCD and NFκB amplification reactions were: 3 min at 94°C, then 1 min at 94°C, 45 seconds at 60°C and 45 seconds at 72°C, repeated 34 times, and at last 5 min at 72°C. For NS1 amplification, the cycles were: 5 min at 94°C, then 45 seconds at 94°C, 30 seconds at 53°C and 1 min at 72°C, repeated 25 times, and at last 10 min at 72°C. All PCR products were confirmed by a single-peak upon melting-curve analysis and by gel electrophoresis. No-template (water) reaction mixtures and “no reverse transcriptase” mixtures were performed on all samples as negative controls.

### Western blot analysis

Proteins were obtained by cell lysis in RIPA buffer (Sigma-Aldrich). Proteins were separated on NuPAGE® Novex 4–12% Bis-Tris gels (Invitrogen-Life Technologies) and transferred on Hybond-PVDF membranes (Amersham) using a Bio-Rad semidry transfer system. Blots were blocked 2 hours at room temperature in 5% nonfat milk in 1% PBS with 0.1% Tween 20. They were next incubated overnight at 4°C with anti-yCD rabbit serum (1/25000, gently provided by Lawrence T.S, University of Michigan), rabbit polyclonal antibody directed against NS1 (1/3000, obtained from INSERM-DKFZ, Heidelberg; Germany), rabbit polyclonal NFκB p65, PARP, Bax and TRAIL antibodies were purchased from Santa Cruz Biotechnology (Tebu-bio, Le Perray en Yvelines, France) and anti-Akt, anti-phospho-Akt (S473) from Cell Signaling Technology (Ozyme, Saint Quentin Yvelines; France). Immunoblots were then developed by the enhanced chemiluminescence (ECL) reagent kit from Amersham-Biosciences (GE Healthcare, Orsay, France), according to the manufacturer's protocol.

### Viability assay

Cells were grown in 96-well plates in 100 µl of medium at a density of 4.10^3^ to 5.10^3^ cells/well, and were infected with virus with or without 5-FC at different concentrations. Cell viability was determined 72 h post infection by MTT assay. After infection and treatment period, media were removed and the cells were incubated in presence of MTT (0.2 mg/ml) at 37°C for three hours. Formazan crystals were next solubilized with 150 µl of dimethylsulfoxyde and absorbance was measured at 570 nm using a microplate reader (Bio-Rad, France). All assays were performed in triplicate, and experiments were repeated three times. Cell death was expressed relative to non-infected and non-treated cells.

### Clonogenic survival assay

Tumor cells were seeded in 24 well plates and forty eight hours following treatment, cells were harvested and 200 to 1000 viable cells (depending on treatment) were plated in triplicate in 60 mm-dishes. Ten to fifteen days later, the number of the formed colonies was determined after Giemsa staining. Briefly, cells were washed with PBS and fixed in methanol/acetic acid for 15 minutes at room temperature. Cells were next incubated at room temperature in a 1% Giemsa solution for 30 minutes, washed with deionized water and air-dried. Each experiment was performed in triplicate.

### Luciferase gene reporter assay

For NFκB transcriptional activity evaluation, cells were transfected with 1 µg of NFκB-luc (DNA plasmids kindly provided by Professor Shinichi Kawai from Toho University School of Medicine, Tokyo, Japan) and further treated with wtPV-H1or rPVH1-yCD plus 5-FC (100 µg/ml), or with MG132 (5 µM) or LY294002 (20 µM). After 48 h, tumor cells were harvested and luciferase assays were performed using the Promega luciferase assay system (Promega, Charbonnières, France) and a luminometer -Lumistar BM6.

### Apoptosis studies

#### Apoptotic cell death analysis by flow cytometry

Cells were infected with the wtPV-H1 or rPV-H1 (10 MOI) with and without 5-FC for 48 h. For the combined effect of virus infection with MG132 (5 µM) or LY294002 (20 µM) inhibitors, the wtPV-H1 and rPVH1-yCD were used at low concentration (1MOI). Thereafter, cells were trypsinized, washed once with PBS and 1.10^6^cells were resuspended in binding buffer and stained with FITC-labeled AnnexinV (AnnexinV-FITC Apoptosis Detection Kit; Roche, France) according to the manufacturer's protocols. To exclude late apoptotic and necrotic cells, propidium iodide (PI, 50 µg/ml) was added to the FITC-AnnexinV stained samples and incubated 30 min at RT. Then, the samples were examined by flow cytometry (FACScan, Becton Dickinson, France) for apoptosis analysis using standard protocol and acquiring 10.000 cells per sample.

#### Caspase activity assays

Cells seeded into 96-well plates (5.000 cells/well) in triplicate were infected and treated 12 hours later. Caspase-3/7 activity was evaluated 48 hours after infection using the Caspase-Glo 3/7 Assay Kit (Promega) according to the manufacturer's instruction.

### Animal tumor model studies

#### Ethics Statement

All procedures involving animals and their care were conducted in compliance with a European Communities Council Directive (2010/63/EU) and under the supervision of authorized investigators. Experimental protocols were reviewed/approved by local ethical comity of Alsace Head Office of the French Department of Veterinary and Public Health Guide for the Care and Use of Laboratory Animals that regulates animal research (*Comité Régional d'Ethique en Matière d'Expérimentation Animale de Strasbourg* (CREMEAS- C2EA35)).

#### Experiments

Female NMRI-nu/nu mice at 5–6 weeks were obtained from Elevage Janvier (Le Genest-St Isle, France) and quarantined for 2 weeks before experiments under controlled environmental conditions with a 12 h/12 h light/dark cycle with an access to Food (UAR-Alimentation) and tap water *ad libitum*. For peritoneal carcinomatosis model, Nude mice were intraperitoneally (i.p) inoculated with AsPc1 tumor cells (10^7^ cells in 1 ml of PBS). Two experimental schedules were designed. In the first, animals were treated 48 h post-tumor inoculation and in the second, 2 weeks later. Mice were randomly divided into four experimental treatment groups (n = 10). The control group received injection of NaCl 0.9%, the three other groups received either i.p. injections of wtPV-H1 (1.10^8^ pfu) or 1.10^8^ RU of rPVH1-GFP or rPVH1-yCD recombinant parvoviruses. Forty eight hours later, mice received i.p injections of 5-FC (250 mg/kg/day) or PBS (control group), daily during two weeks.

#### Transgene expression and tumor growth extension

For mRNA and protein expression analysis, two mice per group from the first experiment were sacrificed 2 and 10 days post-infection; and normal biopsies and tumor nodules were recovered by dissection from various abdominal organs (liver, pancreas, mesentery and abdominal wall). For GFP amplification, specific primers were used, the forward: 5′-taaagggccacaagttcagc-3′ and reverse: 5′-tgttctggtggtagtggtcg-3′ primers. As AsPc1 cells produce the carcinoembryonic antigen (CEA) tumor marker, the tumor growth extension and peritoneal invasion were evaluated by CEA level measurements. Thus, blood samples were collected from the tail vein of mice each 5 days for one month and the CEA concentration was evaluated by ELISA Assay Kit according to manufacturer's instructions (Euromedex, Strasbourg; France). Additionally, animals were observed daily and survival data were analyzed by Kaplan-Meier analysis.

#### PV-H1 and GDEPT/5-FU toxicity studies

To assess the possible toxicity related to PV-H1 infection or systemic 5-FU production, mice were weighted two times weekly during the first three weeks of experiments before development and accumulation of significant ascites fluid in the abdominal cavity.

For PV-H1 toxicity, we evaluated the biodistribution and perseverance of wtPV-H1 and its recombinant derivative expressing yCD in normal tissue compared to tumor nodule tissues. The PV-H1 DNA was extracted using QIAamp DNA mini kit (Qiagen, les Ulis; France) and it was used immediately for quantitative PCR analysis or stored at −20°C. The qPCR measurements were performed with the following primers: sense primer, 5′-TCAATGCGCTCACCATCTCTG-3′ (position nt 1996–2016 within the NS gene region of the PV-H1 genome) and antisense primer 5′-TCGTAGGCTTCGTCGTGTTCT-3′ (position nt 2490–2510). DNA from the PV-H1 plasmid Δ800 served as a positive control. Mouse glyceraldehyde-3-phosphate dehydrogenase (GAPDH) primers (Qiagen) were used as endogenous control for input DNA.

For liver and kidney toxicity, serum samples were collected and used for the measurement of key biochemical marker of their functions including aspartate aminotransferase (ASAT), alanine aminotransferase (ALAT), Creatinine and Urea. The assays were done using commercial kits (Sigma, Courtaboeuf; France) according to the manufacturer's protocol.

#### 5-FU and 5-FC measurement

The level of 5-FU produced following rPVH1-yCD/5-FC was measured 2 days after 5-FC (250 mg/kg/day) i.p administration. Blood samples and tissue biopsies from various organs of control and tumor-bearing mice were collected and used for analysis assessment with high-performance liquid chromatography (HPLC) with UV detection at 269 nm as described in ref. [37]. The same amount of tissues (100 mg) was homogenized and lysed by freezing/thawing three times; thereafter the extracts were centrifuged at 12.000 r/min, 10 min and 4°C. The tissue extracts and plasma were subjected to HPLC analysis.

### Statistical analysis

Mean and standard error of the mean were calculated. The statistically significant difference between treatments was assessed using a one-way analysis of variance followed by a parametric Student unpaired *t* test, as Bartlett's test gave homogeneity of variance. A difference between the values was considered significant when *p*<0.05.

## Results

### Construction of recombinant parvoviruses and transgene expression

**Figure 1:**
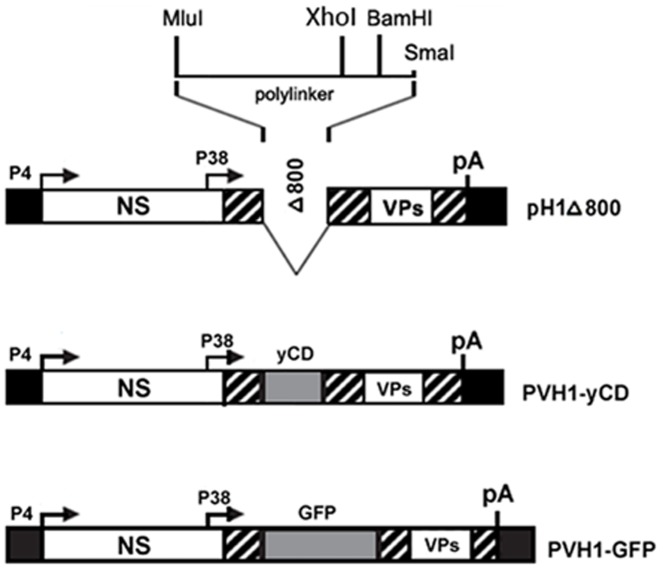
Schematic representation of recombinant parvoviral vector constructions. The upper diagram depicts the empty vector DNA clone PVH1-Δ800 which has 800-bp deletion in the VP coding region and carries a multiple cloning sequence (MluI/SmaI polylinker) at the VP2 translation initiation site. DNA inserts encoding for GFP or catalytic yCD were introduced in polylinker using XhoI and BamH1 restriction enzymes. The resulting rPVH1-GFP and rPVH1-yCD plasmids were used for the production of corresponding non-replicative recombinant parvoviruses.

To examine the possible benefit of a parvovirus vector expressing the suicide gene yCD, we constructed the recombinant H1 virus–based DNA clone rPVH1-yCD, which contains the parvoviral NS genes and the native viral P38 promoter controlling a transgene encoding yCD. As control vector, an identical construct (rPVH1-GFP) was used, which contains the gene encoding GFP marker protein, instead of the yCD gene (Figure 1).

#### Virus infection and transgene expression

**Figure 2:**
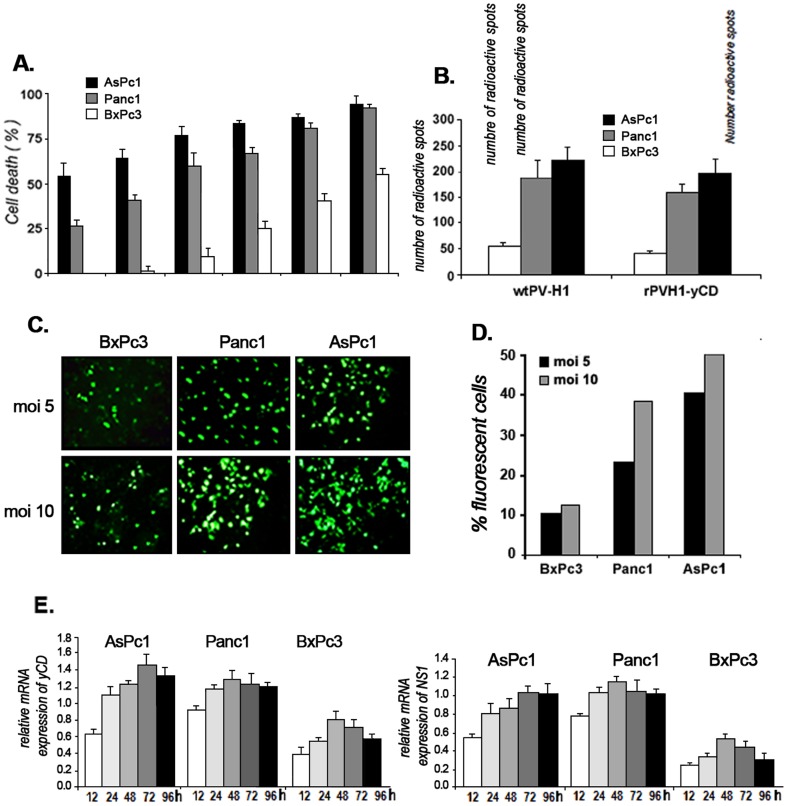
Virus infection and transduction efficiency. **A**) *Cytopathic effect of wtPV-H1*. The tumor cell lines BxPc3, Panc1 and AsPc1 were infected at MOIs ranging from 1 to 100 and after 72 hours, cell viability was measured by MTT assay. Indicated data represent the mean of three independent experiments realized in triplicate. **B**) For pancreatic tumor cell infection study, the cell cultures were blocked in G0/G1 phase by serum starvation for 72 hours and thereafter, tumor cells were infected with wtPV-H1or rPVH1-yCD at MOI 10 for 12 hours, and subsequently transferred on nitrocellulose filters. The detection of NS1 viral gene was realized according to the protocol of PV-H1 titration mentioned above. The detected radioactive spots reflect the number of NS1-hybridized probes. Asterisks indicate significant difference (*** p<0.001) observed between permissive cells (Panc1 and AsPc1) and non-permissive (or low) BxPc3 cells. **C**) Representative microscopy fluorescence images of AsPc1, Panc1 and BxPc3 cell cultures infected with rPVH1-GFP. Cells were infected at MOI 5 and 10 during 48 hours. **D**) Fluorescence intensity was quantified by flow cytometry. Results represent the percentage of fluorescent cells reported to total cells. **E**) For transduction efficiency. Kinetics of NS1 and yCD expression were carried out on AsPc1, Panc1 and BxPc3 cells. Tumor cells were infected with rPVH1-yCD (MOI 10) and total RNA extractions were performed 12, 24, 48 and 72 h post-infection and subjected to real time RT-PCR measurement. Relative quantitation of gene expression was achieved by normalization against the endogenous GAPDH housekeeping gene expression. Error bars represent the standard error of three independent experiments and run in quadruplets.

Because pancreatic adenocarcinomas are diverse in their genetic alterations and histology, we first evaluated the sensitivity of different pancreatic tumor cell lines to the parental wild type PVH1 (wtPV-H1). We observed an MOI-dependent killer effect; the well differentiated and non-mutated KRAS, BxPc3 tumor cells were less sensitive to wtPV-H1 infection than the chemoresistant cell lines AsPc1 and Panc1 (Figure 2A).

Thereafter we verified the capacity of the engineered recombinant rPV-H1 expressing GFP or yCD to infect these tumor cell lines. The cell permissiveness to infection was evaluated by dot blot NS1-hybridization assay. To be sure those results reflect the entry of virus in tumor cells and not its replication; we inhibited the onset of parvovirus multiplication by prevention of the G0/G1 phase transition through serum cell starvation for 72 hours. As indicated in Figure 2B, the infection level obtained with rPVH1-yCD was comparable to wtPV-H1. For functionality, we examined the expression of GFP, NS1 and yCD. The fluorescence microscopy observation revealed that GFP expression was detectable 48 h post-infection. The percentage of fluorescent cells was MOI- and cell line-dependent indicating a variable expression of GFP in the three pancreatic tumor cell lines (Figure 2 C,D). In parallel, using quantitative RT-PCR, we assessed the expression levels of NS1 and yCD mRNAs during 96 hours. Compared to the internal control GAPDH, their expression was time dependent. The expression level of yCD seems to be more important and more sustained than NS1. Despite their low infectivity, BxPc3 cells expressed satisfying levels of NS1 and yCD (Figure 2E). Taken together, these data indicate the correlation between pancreatic tumor cell responsiveness and their permissiveness to wtPV-H1 and rPVH1 generating sufficient expression of NS1 and GFP or yCD.

### The GDEPT system “yCD/5-FC” improves the cytotoxic effect of PV-H1

**Figure 3:**
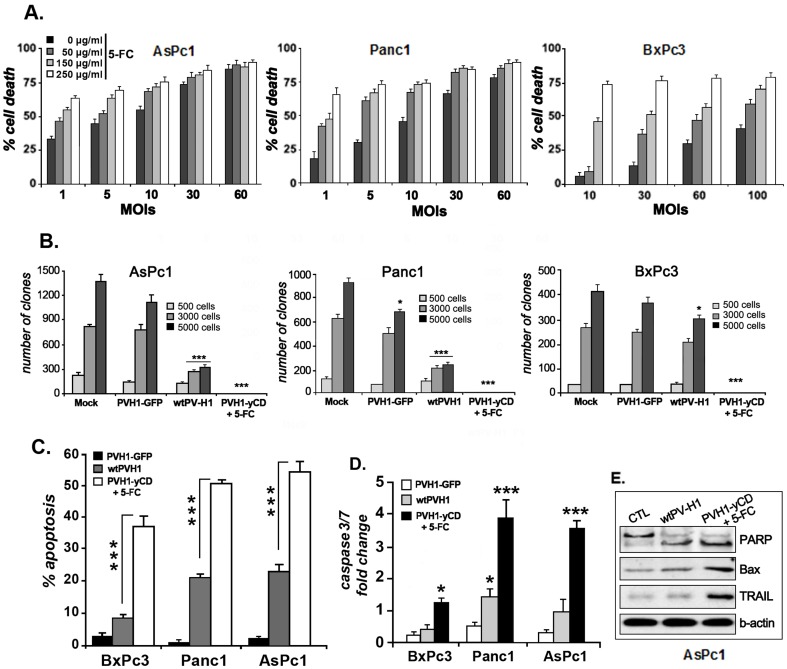
rPVH1 expressing yCD exhibits significant oncolytic effects in human pancreatic tumor cells. **A**) *Cytotoxic effect of rPVH1-yCD/5-FC treatment*. Pancreatic tumor cells were infected with rPVH1-yCD at MOIs (1 to 100) and after 48 hours, cells were either left untreated or treated for three days with different concentrations of 5-FC. Cell survival was assessed by MTT assays and the percentage of cell viability was calculated by comparison to control cell cultures (untreated cells). **B**) *Clonogenic survival assay*. Cells were non-infected or infected with wtPV-H1, rPVH1-GFP or rPVH1-yCD (MOI 10). Cells infected with rH1-yCD were further treated with 5-FC (250 µg/ml) for 48 hours. Then, cells were trypsinized and replated at low density as described in Materials and methods. After 14 days, colony formation was visualized using Giemsa staining and the number of colony was calculated. The number of colony-forming units from non-treated cells was defined as 100% of the surviving fraction. Data represent the mean of three experiments realized in triplicate. Asterisks indicate significant difference observed in PVH1-treated cells compared to untreated cells (mock) (*p<0.05, ***p<0.001). **C**) *Apoptosis induction*. Tumor cells were infected with wtPV-H1 or rPVH1-yCD or rPVH1-GFP (MOI 10). After 48 hours of 5-FC treatment, apoptotic cells were assessed using AnnexinV/PI staining. Results represent mean percentage of apoptotic cells ± s.e.m. of three independent experiments. Asterisks indicate significant difference (***p<0.001) observed in rPVH1-yCD/5-FC treated cells compared to wtPVH1-treated cells. **D**) in parallel, tumor cells were harvested and subjected to caspase3/7 assays. Results represent the average ± s.e.m of three independent experiments and expressed as fold induction compared with untreated control (*p<0.05, ***p<0.001). **E**) Western blot of apoptogene expression after AsPC1 tumor cell infection with wtPV-H1 or PVH1-yCD plus 5-FC treatment. Data indicate expression levels of PARP, Bax and TRAIL compared to the constitutive β-Actin protein. Similar expression profile was observed for BxPc3 and Panc1.

One of the rationales for the construction of the rPVH1-yCD was its postulated ability to improve the wild-type parvovirus cytotoxicity. Following the encouraging reported above data, we performed experiments to evaluate the antitumor activity of the rPVH1 harboring the suicide gene/prodrug system, yCD/5-FC. The cell death level observed after rPVH1-yCD infection increased relative to the MOI and was comparable to that obtained after wtPV-H1 infection (Figure 3A). Further exposition to 5-FC prodrug (250 µg/ml) enhanced the cytotoxicity of rPVH1 (MOI 10) inducing more than 75% (p<0.001) of cell death in AsPc1, Panc1 and BxPc3 cells.

### Clonogenic cell survival assays

To extend the rPVH1-yCD/5FC related *in vitro* cytotoxic effect, colony formation or clonogenic assays were performed in cell cultures. As shown in figure 3B, for tumor cells seeded at high density and after 14 days, the wtPVH1 decreased clonogenic survival in both cell lines AsPc1 and Panc1 by >75% (p<0.001) and in BxPc3 cell line by only 28% (p<0.05). The rPVH1-GFP infection resulted in a less inhibitory effect in cell colony formation assay. Indeed, infection reduced the clonogenic cell survival of AsPc1 and Panc1 by 18% (NS) and 27% (p<0.05), respectively. The survival rate of the low-permissive tumor cells BxPc3 infected with rPVH1-GFP was reduced by only 8% (NS). Interestingly, the most spectacular effect was observed with rPVH1-yCD/5FC which abolished completely the colony formation in Panc1, AsPc1 and BxPc3 tumor cells. Note that the high cytotoxic effect observed in the PVH1-resistant BxPc3cells is related to their high sensitivity to 5-FU produced by the suicide gene/prodrug system, yCD/5-FC. Thus, the viral resistance of this tumor cell line is compensated by its chemosensitivity.

Taken together, these results approve and confirm the MTT data demonstrating clearly that PV-H1 with yCD/5FC arrests the human pancreatic tumor cell growth in chemosensitive and chemoresistant tumor cells.

### The GDEPT system “yCD/5FC” increases PVH1-induced apoptosis

To examine whether the suicide gene/prodrug system enhances PVH1-induced apoptosis, the pancreatic tumor cell cultures were infected with the recombinant rPVH1-yCD, treated with 5-FC (100 µg/ml) and apoptotic cell death was determined after 72 h of treatment. As presented in figure 3C, compared to untreated cells, wtPV-H1 induced 51% and 55% of apoptosis in Panc1 and AsPc1, respectively. This apoptotic effect was significantly augmented with rPVH1-yCD/5-FC treatment by approximately 2.4 folds (p<0.001). For the BxPc3 tumor cells, even they are less permissive for PV-H1 infection; the wtPV-H1-induced apoptosis (9%, NS) was significantly increased by rPVH1-yCD/5FC treatment (4 folds, p<0.001).This remarkable increase could be attributed to the high sensitivity of BxPc3 to 5-FU and the resulting bystander induced effect. Then, we analyzed the activity of Caspase-3 and -7 which are common effector caspases of both intrinsic and extrinsic apoptotic pathways. Our data show that BxPc3 cells infected with wtPV-H1 expressed insignificant level of Caspase3/7, whereas all tumor cells infected with rPVH1-yCD followed by 5-FC treatment generated significant levels of Caspase3/7 (figure 3D). Additionally, we examined the protein expression levels of the proapoptotic gene Bax, TRAIL and PARP in the different tumor cell lines. Our findings indicate a significant expression of these proteins in comparison to the constitutive β-actin protein after infection with PVH1-yCD followed by 5-FC treatment (figure 3E). Data for BxPc3 and Panc1 have the same profile (data not shown).

Together, these observations indicate that yCD/5FC combination improves significantly the antitumor effect of PV-H1 by inhibiting proliferation and increasing apoptosis induction.

### Inhibition of NFκB and Akt/PI3K signaling pathways with rPVH1-yCD/5FC

**Figure 4:**
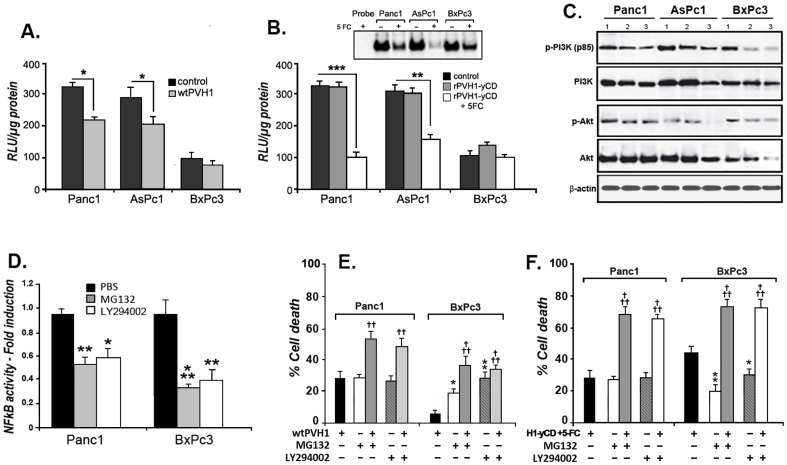
Effect of rPVH1-yCD/5FC treatment on NFκB and Akt/PI3K activity. We first investigated the NFκB constitutive activity in AsPc1, Panc1 and BxPc3 tumor cell lines and compared it to wtPV-H1 (**A**) and rPVH1yCD/5FC (**B**) -infected tumor cells. Thus, tumor cells were transfected with plasmids expressing the luciferase reporter gene, pNFκB-Luc and 24 hours later, cells were infected with wtPV-H1 or rPVH1-yCD. One set of rPVH1yCD-infected cells was further treated with 5-FC. After 48 hours, cells were lysed and luciferase activity was measured using the same amount of protein. Reported data are the mean of three experiments performed in triplicate. The high levels of constitutive NFκB activity observed in AsPc1 and Panc1 were reduced slightly after wtPV-H1 infection (*p<0.05). The rPVH1-yCD/5FC treatment resulted in a significant reduction of constitutive NFκB in Panc1 (**p<0.01) and AsPc1 (***p<0.001). The rPVH1-yCD/5FC data were further confirmed by EMSA experiments. Whole cell extracts were prepared as described in Materials and methods. The specificity of the band corresponding to NFκB-DNA complex was determined using non-labeled NFκB consensus oligonucleotide as a specific competitor. **C**) wtPV-H1 and rPVH1-yCD/5-FC decrease Akt/PI3K constitutive activity. After 48 h of infection and 5-FC treatment, cell lysates were prepared and used to analyze the protein expression of Akt, PI3K and their phosphorylation forms by Western blotting. Compared to the control (1) indicating the high constitutive activity, the pAkt (Ser473) and pPI3K (p85) were strongly inhibited by wtPV-H1 (2) and rPVH1-yCD/5-FC (3). **D**) MG132 and LY94002 reduce significantly the transcriptional activity of NFκB. Data represent the fold induction compared to untreated control cells, *p<0.05, **p<0.01 and ***p<0.001. **E**) NFκB and Akt/PI3K inhibitors (MG132, LY294002) combination enhances the cytopathic effects induced by wtPV-H1 and rPVH1-yCD/5-FC. The indicated mean percentages of cell death are representative of three experiments realized in triplicate. The (†) symbol represents a significant difference between wtPV-H1 or rPVH1-yCD/5-FC treatment alone and their combination with NFκB or PI3K/Akt inhibitors; ††p<0.01, †††p<0.001. The (*) symbol indicates a significant difference between wtPV-H1 or rPVH1-yCD/5-FC treatment and MG132 or LY294002 inhibitors; *p<0.05, **p<0.01.

Constitutive hyperactivation of NFκB transcription factor and Akt/PI3K signaling pathways are a frequent events in cancer associated with chemoresistance, antiapoptotis and increased cell survival. We thus investigated their role in PVH1 oncolytic activity and rPVH1-yCD/5FC-related cell death. As represented in figure 4A, we observed that wtPV-H1 reduce the constitutive activity of NFκB in Panc1 and AsPc1 (p<0.05). However, the chemosensitive BxPc3 presented low constitutive NFκB activity which was not significantly affected by PVH1 transduction. Tumor cells infected with rPVH1-yCD and treated with 5-FC exhibited a significant decrease of NFκB transcriptional activity by 70% (p<0.001) for Panc1 and 48% (p<0.01) for AsPc1. We observed also that this treatment induced substantial inhibition of nuclear NFκB DNA binding activity (figure 4B). We further examined the possible interaction of parvovirus effect and Akt/PI3K signaling pathway. Our data showed that the treatment of pancreatic tumor cell lines with wtPV-H1 and rPVH1-yCD/5-FC resulted in a reduction of the phosphorylation of Akt and PI3K proteins (figure 4C). Thereafter, we determined the impact of NFκB and Akt/PI3K pathways inhibition on virus-induced tumor cell death. At first, we observed that the treatment of Panc1 and BxPc3 with the proteasome inhibitor MG132 (5 µM) and the Akt/PI3K inhibitor LY294002 (20 µM) during 24 hours caused a significant inhibition of NFκB constitutive activity (figure 4D). Then, we examined the cell death effect of these inhibitors alone and in combination with wtPV-H1 and rPVH1-yCD at low concentration (1 MOI). As presented in figure 4E, compared to wtPV-H1, MG132 and LY 294002 alone induced equivalent percentage of apoptosis cell death in Panc1 but significant increase in BxPc3 (figure 4E). For combined treatment, we find that MG132 and LY294002 enhanced respectively the wtPV-H1 cell death effect in Panc1 by 42% (p<0.01) and 45% (p<0.01), and in BxPc3 by 85% (p<0.001) and 83% (p<0.001) (figure 4E). More interestingly, MG132 and LY294002 enhanced rPVH1-yCD/5FC-related apoptosis cell death by 59% (p<0.001) and 57% (p<0.001) in Panc1 and by 43% (p<0.05) and 41% (p<0.05) in BxPc3 (figure 4F). Taken together, these finding strongly suggest that oncosuppressive PVH1 interacts with NFκB and Akt/PI3K signaling pathways. Hence, the wtPV-H1 and rPVH1-yCD/5FC seem to exert their anti-tumor effect on pancreatic tumor cells through an attenuation of NFκB and Akt/PI3K activity.

### “PVH1-yCD/5FC” virotherapy inhibits tumor extension and invasion of pancreatic peritoneal carcinomatosis

Consequent to the improvement of the tumor cell growth inhibition observed *in vitro* after the treatment with rPVH1-yCD/5FC GDEPT system, we sought to assess the usefulness of this new oncosuppressive PV-H1 in peritoneal AsPc1 pancreatic carcinomatosis model.

**Figure 5:**
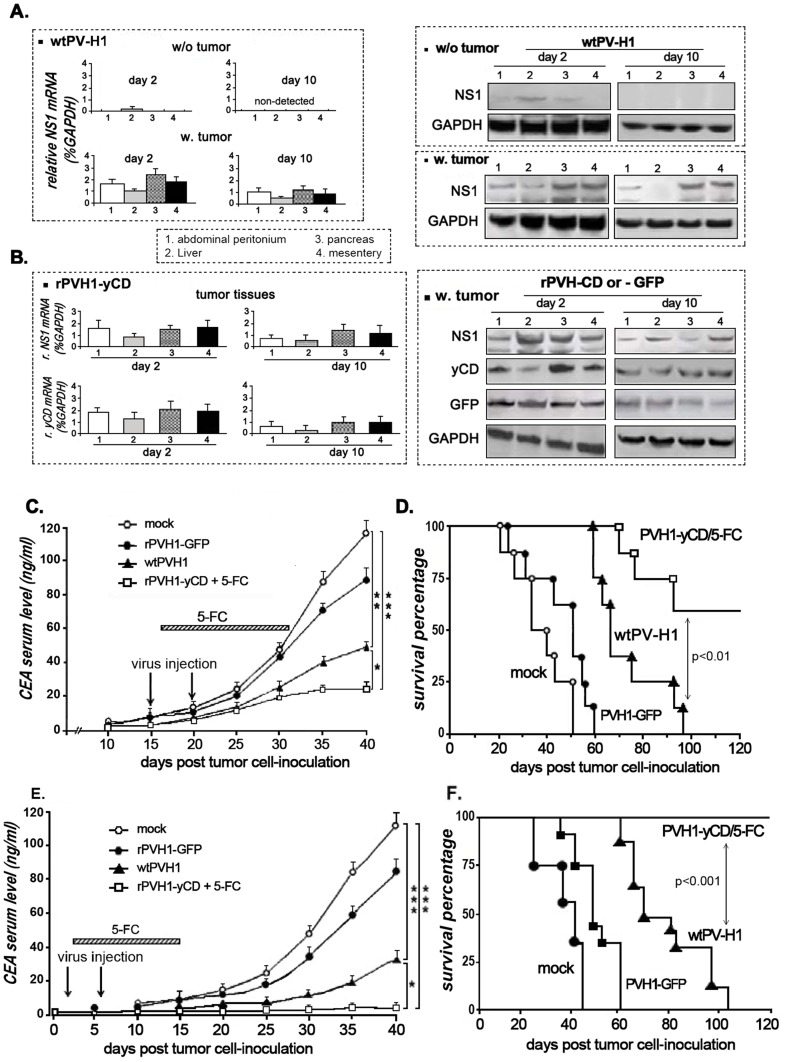
*In vivo* antitumor activity of wild type and GDEPT oncosuppressive parvoviruses on pancreatic peritoneal carcinomatosis AsPc1 tumor model. Mice with peritoneal carcinomatosis (n = 12) received i.p injection of wtPV-H1, rPVH1-GFP or rPVH1-yCD (1.10^8^ particles). Mice treated with rPVH1-yCD also received a daily 5-FC (250 mg/kg/day) i.p administration for 14 days. **A and B**) Real time quantitative RT-PCR measurement and Western blot analysis of NS1, GFP and yCD expression in tumor nodules and normal tissues. For wtPV-H1 infection, two mice per group were sacrificed and relative expression of NS1 was assessed in different organs from tumor-free and tumor-bearing animals. As well, the expression of NS1, GFP and yCD was evaluated on different biopsies arising from tumor-bearing animals injected with either rPVH1-GFP or rPVH1-yCD. GAPDH expression was used as internal control to generate a standard curve. The columns are the mean of three independent experiments; bars, s.e.m. (**C,D**) CEA tumor marker levels and animal survival data of treatment initiated 2 weeks and (**E,F**) 2 days post-tumor cell inoculation. For tumor growth and invasion, studies were performed by tumor maker measurement. Blood samples were drawn every 5 days, and serum CEA levels were measured by ELISA assays (**C,E**). For Kaplan-Meier survival curve determination, mice bearing AsPc1 peritoneal carcinomatosis were monitored for survival for 120 days (**D,F**). Asterisks indicate significant differences observed after PVH1-treated mice compared to non-treated mice (Mock) or rPVH1-yCD/5-FC compared to wtPV-H1; (*p<0.05, **p<0.01 and ***p<0.001).

To follow the distribution and function of the virus after 2 and 10 days of infection, real time RT-PCR and western blot experiments were performed on normal and tumor tissue biopsies to assess NS1 and gene of interest expression. As seen in figure 5A- left panel, in normal tissues (w/o tumor), NS1 mRNA expression was only transiently detected at very low level in the liver biopsy, after the first 2 days of infection with wtPV-H1. However, in mice bearing peritoneal carcinomatosis (w. tumor), NS1 mRNA was expressed in the tumor nodules arising from abdominal wall (1), liver (2), pancreas (3) and mesentery (4) at day 2 as well as at day10. These data were confirmed by Western blot experiments (figure 5A- right panel). For the non-replicative recombinant PVH1, we showed high sustained expression of NS1, GFP and yCD at day 2 and also at day 10 in the four different tumor biopsies (figure 5B).

To follow-up the effect of the virotherapy protocols on the peritoneal carcinomatosis invasion, we checked the level of the CEA tumor marker in blood samples. In the first protocol of treatment and after 40 days (figure 5C), the wtPV-H1 decreased the CEA concentration (49 ng/ml versus 118 ng/ml in the mock group, p<0.01). In the mice treated with rPVH1-yCD/5-FC, the inhibitory effect was more spectacular (22 ng/ml versus Mock, p<0.001). The treatment effect on animal survival was also followed (figure 5D). The median survival for untreated mice was ∼40 days and all mice died by day 51 post-treatment. However, mice treated with oncolytic virus wtPV-H1 had an improved survival compared to the mock group (p<0.01). Interestingly, mice treated with rPVH1-yCD plus 5-FC exhibited a marked enhancement in their overall survival with 60% of mice surviving after four months, which was significantly different from controls (p<0.001) and from wtPV-H1 (p<0.01). Dead mice autopsies revealed massive peritoneal tumor deposits and ascites that were only occasional in surviving mice.

In the second set of experiments, we observed that wtPV-H1 and rPVH1-yCD/5-FC treatment applied 2 days after implantation of tumor cells effectively delays the detection of the tumor marker CEA in the blood. As shown in figure 5E, after 40 days, a significant delay in tumor marker production was observed during 2 to 3 weeks. In rPVH1-yCD/5FC-treated mice, the basal secretion of CEA was at very low level, approximately 2 ng/ml compared to 31 and 152 ng/ml, respectively in wtPVH1-treated group and in control group (figure 5E). Additionally, at autopsy, there were no visible tumor nodules in the peritoneal cavity suggesting an inhibition of the tumor cell anchorage and peritoneal dissemination progression. In accordance with these data, the Kaplan-Meier survival analysis indicates that all mice receiving rPVH1-yCD/5-FC treatment were alive after 120 days (figure 5F). Collectively, these results demonstrate that early 5-FC/yCD parvovirotherapy can delay significantly the progression of the pancreatic peritoneal carcinomatosis in mouse and provide protective benefits against tumor growth and invasion.

### *In vivo* toxicity studies

**Figure 6:**
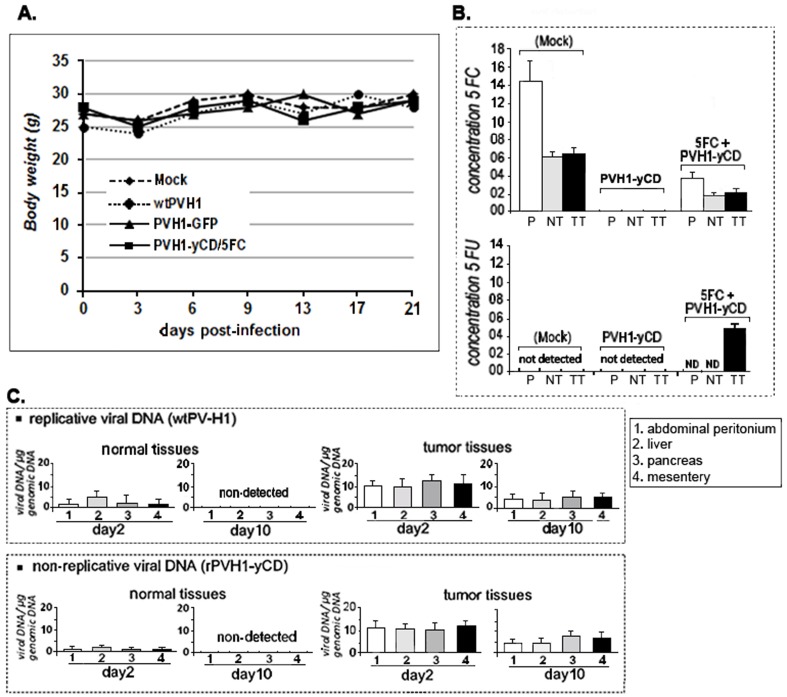
PVH1-yCD/5-FC did not induce significant systemic toxicity in mice. As indicated in **Panel A**, compared to the untreated group (mock), the body weight of mice receiving wtPVH1 or rPVH1-yCD/5-FC was not affected during the first 21 days period of treatment. **Panel B**) the concentrations (µg/ml) of 5-FC and 5-FU were determined using HPLC measurement 48 hours after prodrug i.p injections. In the control group (mock), the concentration of 5-FC in the plasma samples (P) was approximately 14 µg/ml. Its level in normal and tumor tissues (NT, TT) was comparable (6 to 6.25 µg/ml). In the PVH1-yCD/5-FC -treated mice, the levels of 5-FC were inferior (4 µg/ml in plasma samples and 2–2.65 µg/ml in normal and tumor tissue). As indicated in panel B- bottom, in the control group as well as in PVH1-yCD/5FC –treated group, the 5-FU was not detected neither in plasma samples nor in normal tissue. 5-FU (5 µg/ml) was only detected in tumor tissue extracts from the PVH1-yCD/5FC–treated mice. **Panel C**- For viral presence, DNA was extracted and quantitative real-time PCR was done to assess viral copy number per total genomic cellular DNA. The viral DNA was only detected at low level in normal tissue at day 2 after i.p injection of replicative wtPV-H1 or PVH1-yCD. However, in tumor nodule tissues, viral DNA was detected in all the samples (1,2,3,4) at day 2 and 10 post-infection. Columns are the mean of triplicate assays; bars, s.e.m.

To evaluate *in vivo* toxicity of PV-H1/GDEPT system, biochemistry markers of liver and kidney in sera were examined from control and treated mice. Meanwhile, we followed the mice body weight during three weeks after treatment (Figure 6A). As summarized in table 1, the results showed that the liver and kidney function was not impaired in each treatment group. Together, these data indicated that neither serum markers nor body weight of animals differed between virus/5-FC treatment groups versus normal control mice, suggesting that intraperitoneal administration of wPV-H1 or rPVH1-GFP or rPVH1-yCD/5-FC did not cause detectable system toxicity. For hypothetical systemic toxicity related to 5-FC metabolism, HPLC analysis showed clearly no detectable 5-FU in the blood samples (Figure 6B). Interestingly, we demonstrated high local production of 5-FU in tumor nodule tissues 10 days after treatment with rPVH1-yCD/5-FC (Figure 6B). For PV-H1 infection and spreading, the viral DNA was found in tumor tissues infected with wtPV-H1 or rPVH1-yCD after 2 and 10 days (Figure 6C). However, in normal tissues of different organs, the presence of the virus was detectable but only transiently, since no viral DNA was detected 10 days after infection (Figure 6C). Altogether, these data indicate that PVH1-yCD/5-FC protocols did not induce any significant systemic or suspect signs of toxicity.

**Table 1:** Serum analysis for biochemical markers of kidney and liver function in PVH1-yCD/5FC.

treatment	Urea (mmol/L)		Creatinine(µmol/ml)		ALAT (U/L)		ASAT (U/L)	
	2 d	10 d	2 d	10 d	2 d	10 d	2 d	10 d
Mock	4.7±0.3	3.9±0.4	29.3±0.4	30±4.2	29.5±5.7	30.8±3.6	58.1±6.5	63.5±6.2
wtPV-H1	5.1±0.7	4.5±0.6	25.3±0.6	29±3.7	32.0±4.5	27.7±3.2	62.3±7.9	60.2±7.7
PVH1-yCD/5FC	3.2±0.4	4.2±0.5	30.0±0.5	28±4.5	34.3±5.7	31.2±4.1	64.7±7.2	65.4±8.8

## Discussion

Despite availability of advanced medical and surgical treatment, the prognosis of pancreatic cancer remains very poor. Although the development of new chemotherapeutic drugs, there are still significant numbers of relapsed cases, which become chemoresistant. Thus, future effort in identifying and validating different preclinical research will provide new effective therapeutic approaches such as oncolytic virotherapy, particularly for chemoresistant or relapsed patients. In a previous works, we demonstrated that lipofection of suicide gene/prodrug system was obviously efficient to reduce pancreatic tumor carcinomatosis [38]. To increase the efficiency of gene expression and delivery targeting, several groups have experimented with replication-competent viruses. During the last years, PV-H1 and MVMp have been intensively exploited for experimental cancer virotherapy approaches [39], [40]. Although many trials have demonstrated proof of principle and a favorable safety profile, these viruses presented undoubtedly insufficient antitumor efficacy. Therefore, in this study, we designed a recombinant PV-H1 armed with the suicide gene yCD and evaluated its therapeutic potential in preclinical pancreatic peritoneal carcinomatosis model. In one hand, we exploited the natural oncotropic and oncolytic properties of the PV-H1; and in another hand, we wanted to improve its intrinsic cytotoxicity using suicide gene activating prodrug therapy.

The success of tumor virotherapy depends on infection power of tumor cells. Thus, we analyzed the transduction efficiency of the PV-H1 in a panel of pancreatic tumor cell cultures. We found that the different tumor cell lines showed various levels of susceptibility to viral transduction and the cytopathic effect was MOI dependent.

The BxPc3 tumor cells presented the lowest viral transduction and sensitivity as showed by NS1 or GFP expression and cell survival data. The weak rate of virus entry and the reduced expression of NS1 could at least in part explain their lower responsiveness to PV-H1. Indeed, it was reported that oncogenic and tumor suppressor genes influenced greatly the parvovirus replication and transduction [41]. In contrast to Panc1 and AsPC1, the BxPc3 tumor cells are well differentiated with non-mutated K-RAS. Despite the fact that we did not report on the molecular events, our present study revealed further hints concerning the PV-H1 infectivity and oncolytic activity linked to the cell differentiation and genetic abnormalities profiles.

Regarding the recombinant rPVH1-yCD outcome related to the parvoviral NS1 natural toxicity, the cell survival analysis showed a comparable data to that obtained after infection with wtPV-H1. These findings suggest that replacement of VP genes by yCD transgene did not alter the intrinsic properties of PV-H1. Moreover and even in absence of viral replication, rPVH1-yCD infection followed by 5-FC treatment resulted in an increase of the antitumor activity.

Interestingly, the clonogenic survival assay showed that the slightly permissive BxPc3 tumor cells were drastically reduced by rPVH1-yCD/5-FC treatment. This high cytotoxic effect is related to their high sensitivity to 5-FU produced by the suicide gene/prodrug system, yCD/5-FC. In contrast to the chemoresistant tumor cell lines Panc1 and AsPc1, it is well demonstrated by us and other authors that BxPc3 cell line is a sensitive tumor model to chemotherapy [31]–[33]. Thus, the interest of this study is also to prove that this strategy combining chemogene therapy (yCD/5-FC) with oncotropic PVH1 vector represents really an alternative option to overcome the chemoresistant pancreatic tumors. Thus, it is most likely that the combination of PV-H1 with yCD/5-FC system resulted in high production of 5-FU inducing synergistic cytotoxicity with PV-H1 NS1. For the wPV-H1 cytopathic effect, it was reported recently that NS1-induced cytotoxicity is related to DNA damage [42]. So far, the toxicity seen with NS1 wtPVH1-dependent in tumors has been mild compared with that of traditional chemotherapy. Then, we can speculate that the enhanced cytotoxic effect of rPV-H1/yCD is due to 5-FU-mediated inhibition of DNA repair following NS1-induced DNA damage. Moreover, we demonstrated that in addition to the induction of the conventional apoptotic activation of caspase and Bax/TRAIL pathways, the poly(ADP-ribose) polymerase (PARP) was highly activated. This enzyme plays a critical role in the maintenance of DNA integrity and repair. Also, it became a useful hallmark of apoptosis cell death.

Overall, the mechanisms involved in tumor regression during oncolytic therapy are still a matter of heated debate. They may naturally vary among different tumor models and/or be dependent on different oncolytic virus strains used. Thus, in this work, we also wanted to highlight some mechanisms that could be involved in the proposed oncosuppressive rPV-H1/GDEPT virotherapy approach. It was already reported that virus infection and activity are related to a specific recruitment of multiple transcriptional factors and signaling pathways. In the last years, considerable evidence supports the notion that NFκB and PI3K/Akt signaling pathways play critical roles in human cancers promoting tumor cell survival, tumorigenesis and development of pancreatic tumor resistance [22]. The high constitutive NFκB and PI3K/Akt activity is present in the majority of human pancreatic cancer and several pancreatic carcinoma cell lines. This activity is directly correlated with the inhibition of tumor cell sensitivity to apoptosis induced by antineoplastic agents and may thus restrain apoptosis induced by rPVH1 [41]. We demonstrated that parvovirus infection affect slightly the NFκB and PI3K/Akt activity in the different tumor cell lines. An inhibitory effect of NFκB and PI3K/Akt signals was significant mainly in the highly chemoresistant Panc1 and AsPC1 cells after treatment with PVH1-yCD/5FC system. The inhibition of NFκB and PI3K/Akt activity is probably related to a concomitant NS1 and 5-FU cytotoxic effects. The involvement of these signaling pathways in PV-H1 cytocidal effect was furthermore confirmed using their pharmacological inhibitors resulting in a sensitization of the tumor cell lines to wtPV-H1 infection and PVH1-yCD/5FC treatment. These observations provide data on a potential link between oncolytic activity of PV-H1 and NFκB and PI3K/Akt signaling pathways. The fundamental interaction mechanisms of these signal factors in PV infection need further study in the perspective of combined treatment modality for advanced adenocarcinoma with high constitutive activity of NFκB and PI3K/Akt using their specific inhibitors and rPV-H1 expressing suicide genes or proapoptogenes.

To extend the *in vitro* experiments, we performed *in vivo* studies using AsPc1 peritoneal carcinomatosis model to investigate the efficacy of rH1-yCD/5-FC compared to the replicative wtPV-H1. This preclinical mouse tumor model mimics the clinical scenario of human pancreatic cancers, which are known to early develop local invasion and distant spread. We have seen before that these AsPc1 tumor cells are “moderately” sensitive to wtPV-H1; additionally they produce CEA tumor marker which would facilitate to follow-up the efficacy of virotherapy protocols.

For infection and virus distribution, biopsies tested 10 days post-virus i.p injection showed that all tumor nodules were NS1-positive. Indeed, without 5-FC treatment, expression of NS1 and yCD was detected until 21 days post-infection but less important than after day10 (data not shown). At the same time, the normal tissues from pancreas, abdominal cavity, liver and mesentery were negative at day 10.

As for any cancer therapy, the usually late time point of treatment and the survival of low numbers of tumor cells after therapy are generally the reason of non-complete tumor response or frequent relapses. Thus, we designed two experimental virotherapy protocols. In the first, the treatment was started 48 hours post-tumor inoculation and in the second, two weeks after (e.g. advanced disseminated tumors).

The rPVH1-yCD/5FC virotherapy protocol applied at early stage resulted in tumor cell growth eradication and mice cure. The tumor marker, CEA was undetectable and at the autopsy after one month, there was no macroscopic tumor nodule visible. In the meantime, mice group treated with wtPV-H1 revealed elevated plasma levels of CEA after 3 weeks following i.p infection. Our results also show that the replicative wtPV-H1 allowed a significant inhibition of tumor growth and prolongation of survival compared to the control groups. These data are consisting with previous reports confirming the intrinsic oncolytic characteristics of wtPV-H1 [39], [40]. In the second protocol, although no tumors cure, the CEA levels were non-measurable during three weeks after virus injection and 5-FC treatment. Interestingly, even its nonreplicative property, the therapeutic effect of rPVH1-yCD/5FC system was greater than of wtPV-H1. Indeed, we found that rPVH1-yCD/5FC system slows the disseminated peritoneal tumor nodules and prolongs drastically the animal survival time without any significant apparent systemic toxicity. Our study indicated clearly that neither serum markers (for kidney and hepatotoxicity) nor body weight of animals differed between virus/5-FC treatment groups versus normal control mice, suggesting that intraperitoneal administration of wPV-H1 or rPVH1-yCD/5-FC did not cause detectable marker of toxicity. The enhanced therapeutic effect could be related to the local (intratumoral) concentration of 5-FU and thereby inducing a bystander effect. Unlike conventional chemotherapy, the rationale behind PV-H1/GDEPT virotherapy is that, after yCD expression (followed by 5-FC administration) and accumulation of 5-FU into tumor cells, only these cells will die and diffuse passively the produced cytotoxic drug to the neighboring untransduced tumor cells generating strong bystander effect generated by yCD/5-FC. Whatever the mechanism would be, this potent bystander effect is extremely beneficial for the clinical use of this GDEPT virotherapy strategy. Because 5-FU has narrow therapeutic index and systemic dosing result in significant hematological and gastrointestinal toxicities, GDEPT system allows for systemic delivery of an inert nontoxic prodrug (5-FC) and subsequent local (intratumoral) conversion to the active drug (5-FU), thereby limiting systemic toxicity and enhancing cell kill by maximizing local intratumoral cytotoxic drug concentrations.

Collectively, our data demonstrate for the first time that PV-H1 is a valuable expression vector for yCD/5-FC suicide gene/prodrug system. Obviously, the relative intrinsic oncolytic activity was significantly reinforced and enhanced inducing an outstanding cytotoxic activity *in vitro* in pancreatic tumor cell cultures. *In vivo*, convincingly this GDEPT system enhanced the PV-H1 oncolytic activity reducing drastically the peritoneal dissemination of pancreatic carcinoma with considerable prolongation of mice survival. Additionally, when GDEPT parvovirus treatment was applied early (2 days after tumor cell inoculation), there was no tumor growth and all mice were tumor free. This treatment prevents the anchorage and spreading of tumor cells in the abdominal cavity providing protective benefits against tumor growth and invasion. Thus, its combination with surgery can be used to prevent or greatly reduce the potential for recurrence. Regarding the mechanisms of PV-H1 cytoxicity, we observed a potential relevance of NFκB and PI3K/Akt signaling factors in the oncolytic parvovirus function suggesting that their inhibitors could represent a potent option in a combined therapeutic modality for pancreatic cancer treatment. These findings may be essential for rational design of safe and potent GDEPT virotherapy based on PV-H1 for pancreatic cancer treatment.

Overall, our studies demonstrate that the engineered oncotropic oncolytic PV-H1 concomitant with yCD/5-FC gene therapy system is a highly effective strategy for treating advanced pancreatic cancer in human xenograft carcinomatosis model. Moreover, we have shown that this GDEPT parvovirotherapy is particularly interesting because it could be used for the treatment of chemosensitive and chemoresistant tumors.

Finally, given the biological safety, tumor selectivity and antitumor efficacy of this armed PVH1, the strategy “chemogene-virotherapy” merits further investigation as a promising new therapeutic option for metastatic pancreatic cancer. Furthermore, it is increasingly evident that in order to be successful, some type of smart combination therapy will be necessary to have a meaningful impact on advanced cancer.

## References

[1] Ahlgren JD (1996) Epidemiology and risk factors in pancreatic cancer. Semin Oncol 23: 241–250. 8623060

[2] Anderson KE, Potter JD, Mack TM (1996). Pancreatic cancer. In Cancer Epidemiology and Prevention, 2^nd^ ed, eds Schottenfeld D, Fraumani JF, pp. 725–71. Oxford University Press, New York.

[3] Beger HG, Gansauge F, Leder G (2002) Pancreatic cancer: who benefits from curative resection? Can J Gastroenterol 16: 117–120. 11875596 10.1155/2002/174320

[4] Hu JCC, Coffin RS, Davis CJ, Graham NJ, Groves N, et al (2006) A phase I study of OncoVEXGM-CSF, a second-generation oncolytic herpes simplex virus expressing granulocyte macrophage colony-stimulating factor. Clin Cancer Res 12: 6737–6747. 17121894 10.1158/1078-0432.CCR-06-0759

[5] Cattaneo R, Miest T, Shashkova EV, Barry MA (2008) Reprogrammed viruses as cancer therapeutics: Targeted, armed and shielded. Nat Rev Microbiol 6: 529–540. 18552863 10.1038/nrmicro1927 PMC3947522

[6] Guo ZS, Thorne SH, Bartlett DL (2008) Oncolytic virotherapy: Molecular targets in tumor-selective replication and carrier cell-mediated delivery of oncolytic viruses. Biochim Biophys Acta 1785: 217–231. 18328829 10.1016/j.bbcan.2008.02.001 PMC2888475

[7] Nemunaitis J, Ganly I, Khuri F, Arseneau J, Kuhn J, et al (2000) Selective replication and oncolysis in p53 mutant tumors with Onyx-015, an E1B-55kD gene-deleted adenovirus, in patients with advanced head and neck cancer: a phase II trial. Cancer Res 60: 6359–6366. 11103798

[8] Vollmer CM, Ribas A, Butterfield LH, Dissette VB, Andrews KJ, et al (1999) p53 selective and nonselective replication of an E1B-deleted adenovirus in hepatocellular carcinoma. Cancer Res 59: 4369–4374. 10485485

[9] Huang TG, Savontaus MJ, Shinozaki K, Sauter BV, Woo SL (2003) Telomerase-dependant oncolytic adenovirus for cancer treatment. Gene Ther 10: 1241–1247. 12858189 10.1038/sj.gt.3301987

[10] Hernandez-Alcoceba R, Pihalja M, Qian D, Clarke MF (2002) New oncolytic adenoviruses with hypoxia- and estrogen receptor-regulated replication. Hum Gene Ther 13: 1737–1750. 12396626 10.1089/104303402760293574

[11] Anderson MJ, Pattison JR (1984) The human parvovirus. Arch Virol 82: 137–148. 6095787 10.1007/BF01311158

[12] Siegl G (1983) Biology of pathogenicity of autonomous parvovirus. In KI. Berns ed. The parvoviruses. Plenum publishing Corp., New York. pp.297–362

[13] Giese N, Raykov Z, DeMartino L, Vecchi A, Sozzani S, et al (2002) Suppression of metastatic hemangiosarcoma by a parvovirus MVMp vector transducing the IP-10 chemokine into immunocompetent mice. Cancer Gene Ther 9: 432–442. 11961666 10.1038/sj.cgt.7700457

[14] Toolan HW, Saunders EL, Southam GM, Moore AE, Levin AG (1965) H-1 virus viremia in the human. Proc Soc Exp Biol Med 119: 711–715. 14328982 10.3181/00379727-119-30278

[15] Le Cesne A, Dupressoir T, Janin N, Spielmann M, Le Chevalier T, et al (1993) Intralesional administration of a live virus, parvovirus H-1 (PVH-1), in cancer patients: a feasibility study. Proc Ann Meet Am Soc Clin Oncol 12: 297.

[16] Deleu L, Pujol A, Faisst S, Rommelaere J (1999) Activation of promoter P4 of autonomous parvovirus minute virus of mice at early S phase is required for productive infection. J Virol 73: 3877–3885. 10196282 10.1128/jvi.73.5.3877-3885.1999 PMC104165

[17] Takahashi T, Ozawa K, Takahashi K, Asano S, Takaku F (1990) Susceptibility of human erythropoietic cells to B19 parvovirus in vitro increases with differentiation. Blood 75: 603–610. 2404522

[18] Cornelis JJ, Spruyt N, Spegelaere P, Guetta E, Darawshi T, et al (1988) Sensitization of transformed rat fibroblasts to killing by parvovirus minute virus of mice correlates with an increase in viral gene expression. J Virol 62: 3438–3444. 3404581 10.1128/jvi.62.9.3438-3444.1988 PMC253468

[19] - Haag A, Menten P, Van Damme J, Dinsart C, Rommelaere J, et al (2000) Highly efficient transduction and expression of cytokine genes into human tumor cells by means of autonomous parvovirus vectors: generation of antitumor responses in recipient mice. Hum Gene Ther 11: 597–609. 10724038 10.1089/10430340050015789

[20] Huber BE, Austin EA, Richards CA, Davis ST, Good SS (1994) Metabolism of 5-fluorocytidine to 5-fluorouracil in human colorectal tumor cells transduced with the cytosine deaminase gene: significant antitumor effects when only a small percentage of tumor cells express cytosine deaminase. Proc Natl Acad Sci USA 91: 8302–8306. 8058798 10.1073/pnas.91.17.8302 PMC44594

[21] Nyati MK, Symon Z, Kievit E, Dornfeld KJ, Rynkiewicz SD, et al (2002) The potential of 5-fluorocytosine/cytosine deaminase enzyme prodrug gene therapy in an intrahepatic colon cancer model. Gene Therapy 9: 844–849. 12080378 10.1038/sj.gt.3301706

[22] Arlt A, Gehrz A, Müerköster S, Vorndamm J, Kruse ML, et al (2003) Role of NF-κB and Akt/PI3K in the resistance of pancreatic carcinoma cell lines against gemcitabine-induced cell death. Oncogene 22: 3243–3251. 12761494 10.1038/sj.onc.1206390

[23] Montagut C, Tusquets I, Ferrer B, Corominas JM, Bellosillo B, et al (2006) Activation of nuclear factor-kappa B is linked to resistance to neoadjuvant chemotherapy in breast cancer patients. Endocr Relat Cancer 13: 607–616. 16728586 10.1677/erc.1.01171

[24] Santoro MG, Rossi A, Amici C (2003) NF-kappaB and virus infection: who controls whom. Embo J 22: 2552–2560. 12773372 10.1093/emboj/cdg267 PMC156764

[25] Faisst SR, Faisst S, Grangette J, Schlehofe JR, Rommelear J (1993) NFκB upstream regulatory sequences of the HIV-1 LTR are involved in the inhibition of the HIV-1 promoter activity by NS1 proteins of autonomous parvoviruses H-1 and MVMp. Virol 197: 770–773. 10.1006/viro.1993.1654 8249299

[26] Pham CG, Bubici C, Zazzeroni F, Knabb JR, Papa S, et al (2007) Upregulation of Twist-1 by NF-{kappa}B blocks cytotoxicity induced by chemotherapeutic drugs. Mol Cell Biol 27: 3920–3935. 17403902 10.1128/MCB.01219-06 PMC1900008

[27] Pikarsky1 E, Porat RM, Stein I, Abramovitch R, Amit S (2004) NF-κB functions as a tumour promoter in inflammation-associated cancer. Nature 431: 461–466. 15329734 10.1038/nature02924

[28] Karin M, Yamamoto Y, Wang QM (2004) The IKK NF-kappa B system: a treasure trove for drug development. Nat Rev Drug Discov 3: 17–26. 14708018 10.1038/nrd1279

[29] Luo JL, Kamata H, Karin M (2005) IKK/NF-kappaB signaling: balancing life and death–a new approach to cancer therapy. J Clin Invest 115: 2625–32. 16200195 10.1172/JCI26322 PMC1236696

[30] Mueller A, Bachmann E, Linnig M, Khillimberger K, Schimanski CC, et al (2012) Selective PI3K inhibition by BKM120 and BEZ235 alone or in combination with chemotherapy in wild-type and mutated human gastrointestinal cancer cell lines. Cancer Chemother Pharmacol 69: 1601–15. 22543857 10.1007/s00280-012-1869-z

[31] Shi X, Liu S, Kleeff J, Friess H, Büchler MW (2002) Acquired resistance of pancreatic cancer cells towards 5-Fluorouracil and gemcitabine is associated with altered expression of apoptosis-regulating genes. Oncology 62: 354–62. 12138244 10.1159/000065068

[32] Melisi D, Xia Q, Paradiso G, Ling J, Moccia T, et al (2011) Modulation of pancreatic cancer chemoresistance by inhibition of TAK1. J Natl Cancer Inst 103: 1190–204. 21743023 10.1093/jnci/djr243 PMC3149044

[33] Réjiba S, Bigand C, Parmentier C, Hajri A (2009) Gemcitabine-Based Chemogene Therapy for Pancreatic Cancer Using Ad-dCK::UMK GDEPT and TS/RR siRNA Strategies. Neoplasia 11: 637–650. 19568409 10.1593/neo.81686 PMC2697350

[34] Kestler J, Neeb B, Struyf S, Van Damme J, Cotmore SF, et al (1999) Cis requirements for the efficient production of recombinant DNA vectors based on autonomous parvoviruses. Hum Gene Ther 10: 1619–1632. 10428207 10.1089/10430349950017626

[35] Kestler J, Neeb B, Struyf S, Van Damme J, Cotmore SF, et al (1999) Cis-requirements for the efficient production of recombinant DNA vectors based on autonomous parvoviruses. Hum Gene Ther 10: 1619–1632. 10428207 10.1089/10430349950017626

[36] Maxwell IH, Maxwell F (1994) A modified plaque assay and infected cell hybridization assay for wild-type and recombinant LuIII autonomous parvovirus. Biotechniques 16: 876–881. 8068342

[37] Nassim MA, Shirazi FH, Cripps CM, Veerasinghan S, Molepo MJ, et al (2002) An HPLC method for the measurement of 5-fluorouracil in human plasma with a low detection limit and a high extraction yield. Int J Mol Med 10: 513–516. 12239603

[38] Hajri A, Wack S, Lehn P, Vigneron JP, Lehn JM, et al (2004) Combined suicide gene therapy for pancreatic peritoneal carcinomatosis using BGTC liposomes. Cancer Gene Ther 1: 16–27. 10.1038/sj.cgt.7700628 14681723

[39] Dupressoir T, Vanacker JM, Cornelis JJ, Duponchel N, Rommelaere J (1989) Inhibition by parvovirus H-1 of the formation of tumors in nude mice and colonies in vitro by transformed human mammary epithelial cells. Cancer Res 49: 3203–3208. 2541900

[40] Faisst S, Guittard D, Benner A, Cesbron JY, Schlehofer JR, et al (1998) Dose-dependent regression of Hela cell-derived tumors in SCID mice after parvovirus H1 infection. Int J Cancer 75: 584–589. 9466660 10.1002/(sici)1097-0215(19980209)75:4<584::aid-ijc15>3.0.co;2-9

[41] Rayet B, Lopez-Guerrero JA, Rommelaere J, Dinsart C (1998) Induction of programmed cell death by parvovirus H-1 in U937 cells: connection with the TNFa signalling pathway. J Virol 72: 8893–8903. 9765434 10.1128/jvi.72.11.8893-8903.1998 PMC110306

[42] Hristov G, Krämer M, El-Andaloussi JN, Mora R, Daeffler L (2010) Through Its Nonstructural Protein NS1, Parvovirus H-1 Induces Apoptosis via Accumulation of Reactive Oxygen Species. J Virol 84: 5909–5922. 20375165 10.1128/JVI.01797-09 PMC2876649

